# Spatial proliferation of African swine fever virus in South Korea

**DOI:** 10.1371/journal.pone.0277381

**Published:** 2022-11-07

**Authors:** Shraddha Tiwari, Thakur Dhakal, Ishwari Tiwari, Gab-Sue Jang, Yeonsu Oh

**Affiliations:** 1 Department of Veterinary Pathology, College of Veterinary Medicine and Institute of Veterinary Science, Kangwon National University, Chuncheon, Republic of Korea; 2 Department of Life Science, Yeungnam University, Gyeongbuk, Republic of South Korea; 3 Department of Anatomy, Physiology and Biochemistry, Agriculture and Forestry University, Chitwan, Nepal; Institute of Advanced Materials, IAAM, SWEDEN

## Abstract

The African swine fever virus (ASFV) was first detected in South Korea on a pig farm in September 2019. Despite active preventive measures to control the spread of ASFV, outbreaks on pig farms and in wild boar have been increasing. In this study, we investigated the spatial contamination area using the minimum convex polygon (MCP) approach, and growth rate using a logistic diffusion model. On the basis of the ASFV outbreak locations recorded from September 17^th^, 2019, to May 20^th^, 2022, the MCP area for the second week was 618.41 km^2^ and expanded to 37959.67 km^2^ in the final week. The maximum asymptote of the logistic function was considered as the land area of South Korea, and we estimated logistic growth rates of 0.022 km^2^ per week and 0.094 km^2^ per month. Administrative bodies should implement preventive and quarantine measures for infectious diseases. The results of this study will be a reference for epidemiologists, ecologists, and policy makers and contribute to the establishment of appropriate quarantine measures for disease control and management.

## Introduction

African swine fever virus (ASFV) has been categorized as the most severe animal disease (with a mortality of approx. 100% in domestic pigs) that the world has faced in a long time [1]. ASFV was first recorded in Kenya in 1921 [2], and it has been spreading throughout Europe and Asia since its first report in Georgia in 2007 [3, 4]. In South Korea, the first ASFV case was recorded on September 16^th^, 2019 [5], and approximately 2632 cases (21 on domestic pig farms and 2611 in wild boars) were reported between October 9^th^, 2019, and May 20^th^, 2022 [6, 7]. Globally, ASFV poses a significant threat to the swine industry, owing to its epidemiological behavior and current spread in both wild boar and domestic pig populations [8]. To control the spread of ASFV, eradication programs, based on the rapid diagnosis, disposal, and slaughter of all animals in an infection zone, thorough cleaning and disinfection, surveillance, desensitization, and movement control measures, should be applied [9, 10]. Ecologists have suggested that wild boar [11], wolves [12], ticks [13], synanthropic birds [14], feed houses [14], slaughterhouses [15], and wildlife in general [16] play a prominent role in the spread, infection, and maintenance of ASF [17, 18]. The direction of epidemic waves can be observed and predicted based on disease vector habitats, movement range, and movement patterns [17, 19]. In the present study, we adopted a minimum convex polygon (MCP) approach to track the infectious boundary zone [20].

The boundary range for wildlife was conceptualized in the early 20^th^ century [21–23], and over the years, techniques of increasing sophistication and complexity have been introduced [24–28]. MCP analysis is an internationally accepted standard method for examining a range of species, particularly in circumstances with presence-only spatially explicit data [29]. The MCP is the smallest area with all occurrence points and no acute angles at its boundary [30]. Although in Korea, active preventive measures have been adopted, the spatial distribution of ASFV outbreaks has been increasing continuously since ASFV was first confirmed at a pig firm in Paju-si, Gyeonggi-do, northern South Korea, on September 16^th^, 2019 [6, 7, 31].

The lack of vaccinations and effective treatment methods make the control and prevention of ASFV particularly challenging [31]. Disease occurrence locations and buffer zones must be continuously recorded [31, 32], and epidemiologists need to rapidly assess the presence of wildlife species in control areas and identify potential vectors to minimize the risk of disease transmission [33]. To assist in disease management goals, we examined the contaminated minimum convex boundary and estimated the weekly, monthly, and annual increment rates, assuming the maximum spread area as the convex polygon of outbreak locations using a widely applied logistic growth function [34].

## Materials and methods

### Study area

**Fig 1:**
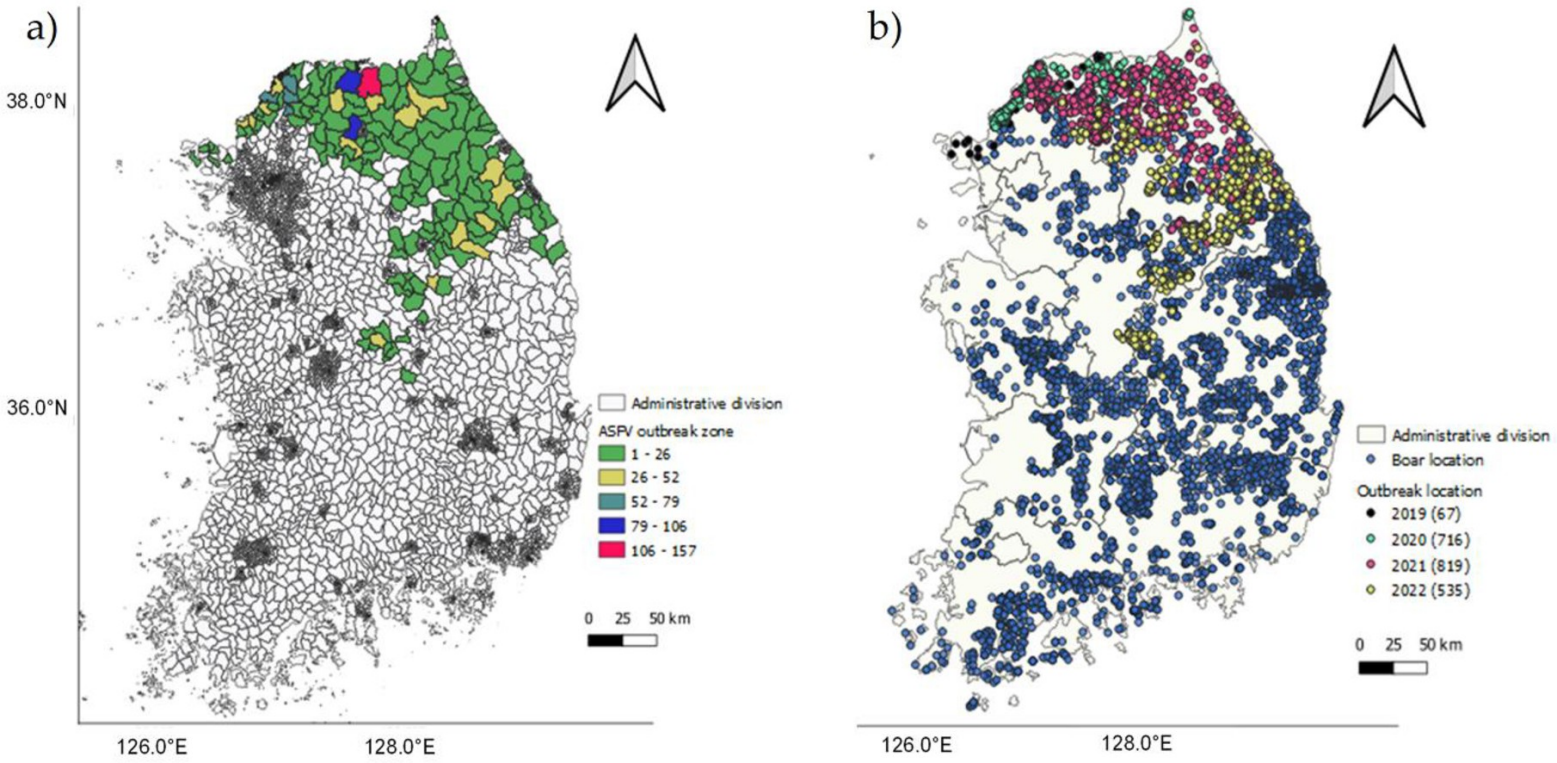
Survey area maps. (a) ASFV outbreak frequency in local administrative areas, and (b) ASFV outbreak and reported locations of wild boar in provincial administrative areas analyzed using QGIS version 3.24.1. Authors specify that this figure is licensed under CC BY 4.0.

South Korea is an East Asian country (latitudes 33° and 39°N, and longitudes 124° and 132°E), located in the southern part of the Korean Peninsula, sharing a land border with North Korea in the north. It has a land area of 100,266 km^2^ [35] and a population of approximately 51.75 million [36]. The country has a diverse climatic range, high precipitation, and complex terrain, which provides suitable habitats for a heterogeneous range of wildlife (with more than 100,000 species of animals and plants being recorded) [37]. The ASFV high-risk wild boar is one of the LR/lc (lower risk/least concern) animals among the 127 mammalian species (84 terrestrial and 43 marine) found in South Korea [38]. Fig 1 presents choropleth maps of the study area, showing the ASFV outbreak frequencies in local administrative areas [39] (Fig 1A) and the outbreak locations in the different years from September 17^th^, 2019, to May 20^th^, 2022, with the recorded occurrence locations of wild boar surveyed between 2014 and 2018 [40] in provincial administrative areas (Fig 1B).

### Data

**Fig 2:**
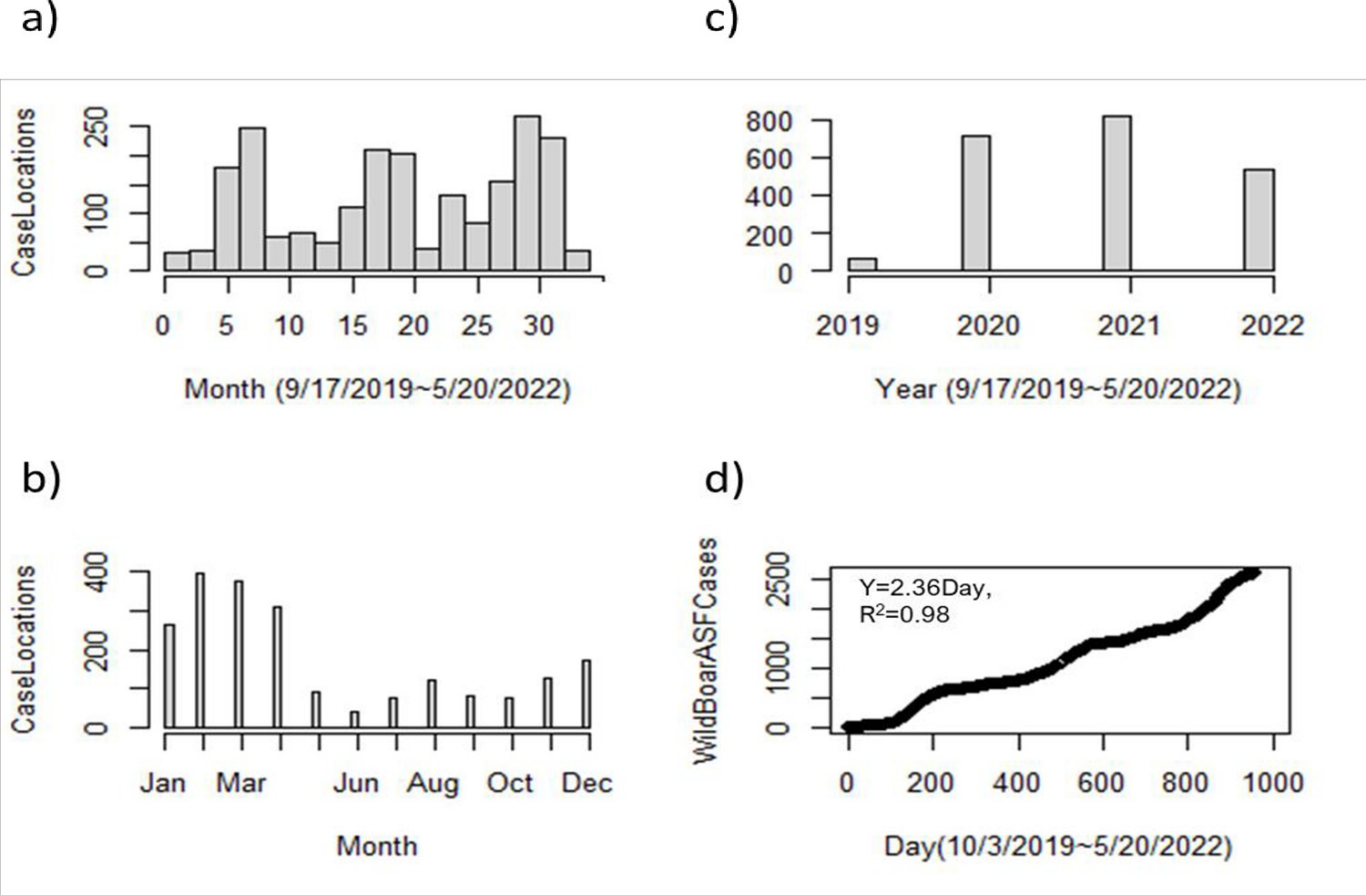
ASFV outbreak locations and cases in different time windows. (a) A frequency plot of ASFV records in 33 months, (b) a frequency plot with respect to months (Jan to Dec), (c) a frequency plot with respect to year since the first confirmed case, and (d) the daily growth of ASFV cases in wild boar since October 3^rd^, 2019 to 20 May 20^th^, 2022 (1275 days).

Data and information pertaining to ASFV outbreaks between September 17^th^, 2019, and May 20^th^, 2020, were mined from the World Organization for Animal Health (OIE) based Pigpeople [6, 7] portal. During the observation period, 2632 cases were reported (21 in domestic pig farms and 2611 in wild boar) in 2137 locations. When the outbreak locations were mapped according to local administrative areas, Hwacheon Gun was established to have highest frequency of outbreaks (157), followed by Sangseomyeon (93), Seomyeon (79), and Yeoncheon (71) (Fig 1A). The total analyzed ASFV case locations in 2019, 2020, 2021, and 2022 were 67, 716, 819, and 535, respectively (Figs 1B and 2C, and Table 1). The details of the data on outbreak frequencies in different timeframes and the increase in ASFV cases in wild boar (Fig 2D) are shown in Fig 2. Higher frequencies of ASFV outbreak were detected in the first quartile of the year, with the highest frequency occurring in February (396 outbreaks), followed by March (378) and April (308) (Table 1).

**Table 1:** ASFV outbreak locations in South Korea during different time periods with quartile breakdown.

Year	2019		2020				2021				2022		Total
Quarter Month	3	4	1	2	3	4	1	2	3	4	1	2	
Jan			66				80				117		263
Feb			115				129				152		396
Mar			149				94				135		378
Apr				100				111				97	308
May				40				19				34	93
Jun				19				20					39
Jul					30				45				75
Aug					37				85				122
Sep	9				26				49				84
Oct		22				23				33			78
Nov		14				52				63			129
Dec		22				59				91			172
**Total**	**9**	**58**	**330**	**159**	**93**	**134**	**303**	**150**	**179**	**187**	**404**	**131**	**2137**

On the basis of monthly outbreaks, we identified three well-defined waves (peaks), at approximately 0–10, 11–21 and 22–33 months (Fig 2A), with higher case numbers being reported in January, February, March, and April (Fig 2B). The cumulative ASFV cases (Total 2611) in wild boar during the study period also increased continuously since initially being detected on October 3^rd^, 2019 (Fig 2D), with a linear growth pattern (Cum. Cases = 2.36×Day, R^2^ = 0.98).

### Model

To determine the spatial proliferation of ASFV, we divided the data into weekly (139 weeks), monthly (33 months), and annual (4 years) timeframes, and the MCP (100%) of each unit timeframe was calculated using the animal home estimation tool ’adehabitatHR’ V. 0.4.19 [41] in the RStudio environment (V. 4.1.3) [42]. A minimum of five coordinates are required to construct one complete polygon [41], and given that there were only three coordinates in the first week of the study period, the data collected during this period were excluded from the analysis. The set of areas (*A*) in Eq 1 was further assessed using a logistic model to analyze the growth pattern in each unit timeframe (*t*) of *the n*^th^ period.


$$A=\{a_{t},a_{t+1},a_{t+2},..a_{t+n-1}\}\;\forall t,n>0$$ (1)

The standard form of the logistic differential function (Eq 2) [43] and the root (Eq 3) were considered to fit the expansion of the contamination zone. The growth parameters were estimated using the non-linear least squares (nls command) method of the R-studio platform [42]. Fitting with non-linear least squares, necessitates initial start parameters [44], which were obtained from the linear model (intercept and time) using logit transformation and the scaling area via a reasonable initial approximation of the asymptote (100,000 km^2^).

$$\frac{df(t)}{dt}=f(t)(1-f(t)$$ (2)

$$f(t)=E(A=K|t)=\frac{K}{1+e^{-(\alpha +\lambda t)}},$$ (3)

where *f(t)*, *K*, *α*, *λ*, and *t* are the logistic growth function, maximum asymptote (maximum virus coverage range), displacement parameter along the time axis, logistic growth rate, and time, respectively. Given that wild boar have spread throughout the country (see Fig 1B), the maximum asymptote (*K*) in the model was considered as the total land area of South Korea (100,266 km^2^). Finally, to evaluate the model performance for each timeframe, we applied the extensively used R-square error (*R*^*2*^) (Eq 4) and mean absolute percentage error (MAPE) metrics (Eq 5). The value *R*^*2*^, which ranges from 0 to 1, is the error coefficient that provides an information; of how well the data fit the original data. Higher values of both R^2^ and MAPE are interpreted as indicating models with a better fit and greater predictive capability [45]. When *X*_*t*_ is the predicted and *Y*_*t*_ is the actual observed *t*^*th*^ value of *n* observations, R2 and MAPE are mathematically calculated as follows:

$$R^{2}=1-\frac{\sum_{t=1}^{n}{\left(X_{t}-Y_{t}\right)}^{2}}{\sum_{t=1}^{n}{\left(\bar{Y}-Y_{t}\right)}^{2}}$$ (4)

$$MAPE=\frac{100}{n}\sum_{t=1}^{n}\left|\frac{{(Y}_{t}-X_{t})}{Y_{t}}\right|,$$ (5)

where $\bar{Y}$ is the average of the actual observation data.

## Results

**Fig 3:**
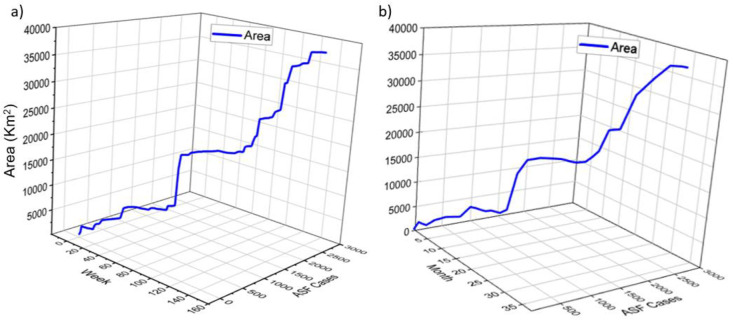
The growth of ASFV cases and polygon size (km^2^) on a (a) weekly and (b) monthly basis.

To examine the minimum possible infected zone using the MCP approach, we performed weekly, monthly, and annual analysis of data relating to ASFV outbreak locations during the survey period. Polygon size and ASFV case numbers were found to increase at each of the assessed time points (Fig 3). An example of the monthly growth of ASFV cases and MCP areas is illustrated in a glyph star plot (see S1 Appendix), which is designed to visualize the pattern of multivariate data involving more than two features [46, 47]. To construct the plot, we used the monthly data with the corresponding two attributes, MCP area and cumulative ASFV outbreaks.

The convex polygon area was 618.41 km^2^ in the first month and increased to 37,959.68 km^2^ at the end date of the survey period on May 20^th^, 2022.

In Table 2, we present the estimated logistic parameters of the time series growth of the area in weekly, monthly, and annual timeframes, considering the maximum asymptote as the total land area (100,266 km^2^) of the nation, using the non-linear least square regression approach.

**Table 2:** Estimated logistic growth parameters.

	Parameters	Estimate	Std. error	t-value	Pr (>|t|)	R^2^ (MAPE)
Week (*N* = 139)	*a*	-3.532***	0.063	-56.340	0.000	0.948 (21.622)
	λ	0.022***	0.001	38.690	0.000	
Month (*N* = 33)	*a*	-3.575***	0.128	-28.020	0.000	0.955 (29.316)
	λ	0.095***	0.005	19.720	0.000	
Year (*N* = 4)	*a*	-3.340*	0.491	-6.808	0.021	0.958 (52.962)
	λ	0.726*	0.143	5.084	0.037	

*** and ‘

* denote significant values at the *p* < 0.001 and *p* < 0.05 levels.

The displacement factor (*a*) from the time axis and growth rate (λ) for the weekly, monthly, and annual timeframes were (-3.532, 0.022), (-3.575, 0.095), and (-3.340,0.726), respectively. We detected highly significant fits (p < 0.001) for weekly and monthly data with R^2^ and MAPE values of 0.948, and 21.622% and 0.955 and 29.316%, respectively, whereas for the annual data, we detected significance at the p < 0.05 level, with a higher MAPE value (52.96%). On the basis of the MAPE errors and their level of significance, we established that the weekly growth model provided a better fit than the monthly or annual model (Table 2).

**Fig 4:**
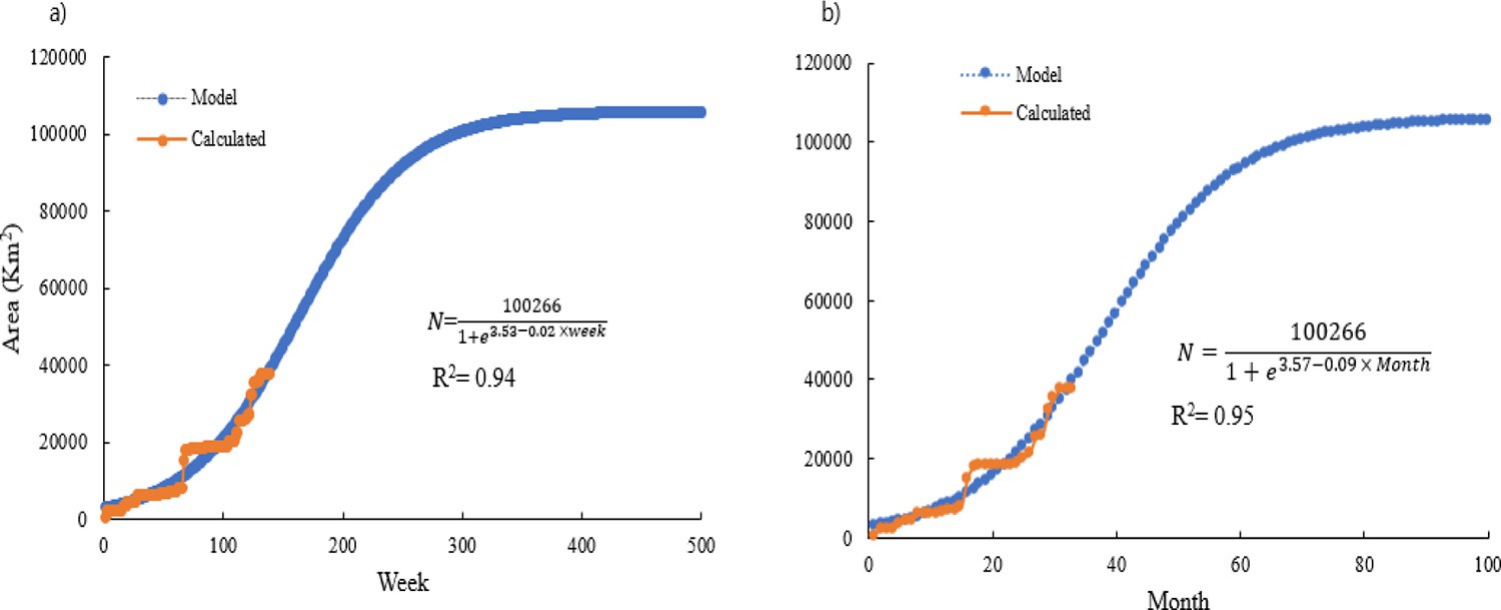
ASFV infection growth boundary areas and logistic fit under (a) weekly and (b) monthly scenarios.

The findings of our reproduction of the data based on the estimated logistic growth parameters indicated that growth stabilizes at approximately 300 weeks and 70 months from the initial outbreak. Details of the spatial growth curves are shown in Fig 4.

## Discussion and conclusion

The continuous circulation of ASFV and expansion in the distribution of reported cases, particularly in forests and mountainous areas, pose a significant threat to the swine industry and wildlife [15, 48]. In the present study, we analyzed the spatial growth of ASFV based on 2623 wild boar and 21 pig farm cases reported from 2137 locations in South Korea during the period from September 17^th^, 2019, to May 20^th^, 2022. Wild boar are considered a key factor in the spread and management of ASFV [16, 49–51], and on the basis of survey data collected between 2014 and 2018 [40], wild boars have extended their range throughout the nation (see Fig 1B). In this study, we considered the maximum asymptote (carrying capacity) as the total land area (100,266 km^2^), and proposed an epidemic spatial proliferation model based on the logistic growth curve using the MCP approach.

The first case of ASF in South Korea was detected in Paju-si, Gyeonggi-do, near the border with North Korea, approximately 4 months after a reported outbreak in Northern Pyongan-namdo Province (May 30^th^, 2019) [7, 31, 52]. The persistence of ASFV outbreaks in the Russian Federation, Europe, China, and other Asian countries, including Korea, has raised awareness of the detrimental impact of this virus on the global pork and food processing industries. South Korea has been implementing active control measures to eradicate the virus by identifying the biosafety risks associated with movements of people, vehicles, and boar; destroying pig herds; swill feeding of wild boar; handling of wild boar during hunting and trapping; and disposing of and searching for carcasses [31]. However, despite the training of wild boar capture and professional carcass search teams, biosafety procedures are sometimes ignored, and given the limitations of the applied measures, including fencing and trapping [31], the cases and spatial distribution of ASFV have continued to gradually increase from the northern to southern region of South Korea (Figs 1 and 2).

Wild boar outbreaks and MCP areas are impacted with locations and seasonals (see Fig 2). During the mating season between October and May, boars come out into heat, and with dispersal and group formation, there is an increase in the frequency of interactions [53, 54], which could be a possible factor contributing to the higher number of outbreaks during this period (Figs 2B and 3).

In the present study, we applied MCPs to analyze the weekly, monthly, and annual spatial growth of ASFV. Simple graphs of locational data can reveal significant information [53, 54]. The logistic growth parameters of MCP areas in the current models for the weekly, monthly, and annual timeframes were (-3.532, 0.022), (-3.575, 0.095), and (-3.340, 0.726), respectively, and we established that the analysis of micro-level data on a weekly basis yielded more significant information with a better fit than either the monthly or annual models (see Table 2 and Fig 3). By gaining an understanding of growth patterns in this way, plans for disease spread management and the establishment of buffer zones can be suitably modified [55].

We believe that this study provides a reasonable macro-level analysis of the spatial proliferation of ASFV in special cases, such as in South Korea, in which the outbreak locations expanded progressively from north to south. However, we did not assess growth within local boundaries or the factors contributing to virus transmission. Among the numerous epidemic diffusion models available [56–58], populations with spatial growth could be analyzed and validated, including the use of logistic functions, in future studies. The maximum asymptote on logistic fit was designed based on a consideration of the possible spread area of disease vectors (wild boar), land cover features, and barriers to vector movement that may influence the diffusion pattern, and by taking into account such factors and the activities of other vectors such as birds and ticks, local and global scenarios could be analyzed. In addition, further studies could examine details relating to the habitat suitability of disease hosts (determined using tools such as MaxEnt) [59], minimum-volume ellipsoid (MVE) [60], and wildlife corridors [61], which could be applied with occurrence data. Moreover, information on the pattern of infection cases could provide a basis for research on disease control and management strategies.

To the best of our knowledge, there are significant studies on the disease growth rate and basic reproduction number in suidae [57, 62–67] but spatial growths have not considered. In the present study, we applied an approach to analyze the spatial proliferation of ASFV in South Korea based on logistic growth parameters. Given the current lack of vaccine against ASFV, the eradication of disease vectors, control, and prevention are the main methods for constraining the spread of ASFV [68]. In this regard, it will be beneficial to assess disease dynamics and precautionary measures against viruses, and to gain better understanding of the spatial extent of the area of contamination and growth rate. Although no model is perfect, most are useful to varying extents [69], and in the present study, we consulted veterinary inspectors and scientists regarding the proposed method, and accordingly believe that this model could serve as a supportive tool in the establishment of policies for wildlife disease management.

## Supporting information

S1 Appendix A glyph star plot of cumulative area and ASFV cases in each indexed month (total, 33 months). The shape of each glyph increases with time (months), and the area of minimum convex polygons and ASFV cases increases continuously. The glyph plot imposes regularity on the variation and thereby enables a clear visualiztion of the monthly growth patterns.
(DOCX)
