# Supplementary material for: Spatial proliferation of African swine fever virus in South Korea
Source: PLoS One. 2022 Nov 7;17(11):e0277381. doi: 10.1371/journal.pone.0277381 (PMC9639837; doi:10.1371/journal.pone.0277381)

**S1 Appendix**

A glyph star plot of cumulative area and ASFV cases in each indexed month (total, 33 months). The shape of each glyph increases with time (months), and the area of minimum convex polygons and ASFV cases increases continuously. The glyph plot imposes regularity on the variation and thereby enables a clear visualiztion of the monthly growth patterns.


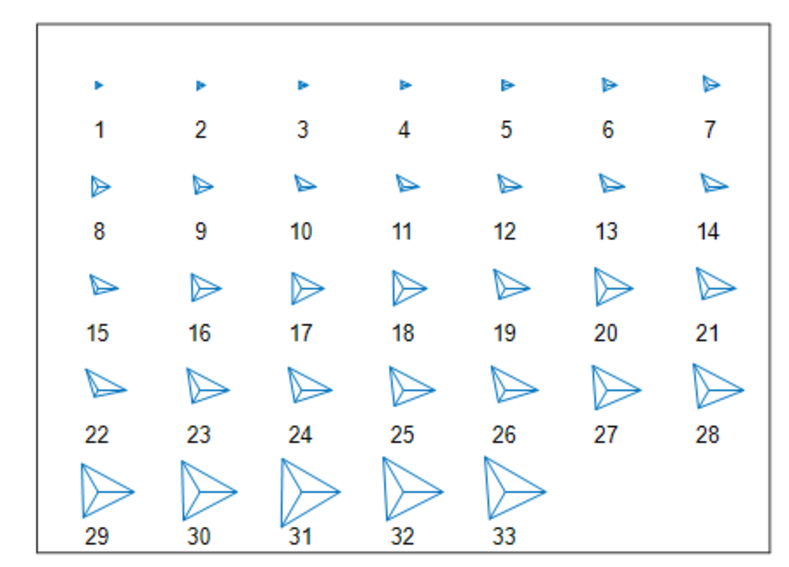

Supplement: S1 Appendix — The shape of each glyph increases with time (months), and the area of minimum convex polygons and ASFV cases increases continuously. The glyph plot imposes regularity on the variation and thereby enables a clear visualiztion of the monthly growth patterns. (DOCX) [file pone.0277381.s001.docx]
